# Diversity, Distribution, and Resistance Profiles of Bacterial Bloodstream Infections in Three Tertiary Referral Hospitals in Rwanda Between 2020 and 2022

**DOI:** 10.3390/antibiotics13111084

**Published:** 2024-11-14

**Authors:** Misbah Gashegu, Vedaste Ndahindwa, Edson Rwagasore, Albert Tuyishime, Clarisse Musanabaganwa, Noel Gahamanyi, Isabelle Mukagatare, Djibril Mbarushimana, Christopher Aird Green, Tafadzwa Dzinamarira, Ayman Ahmed, Claude Mambo Muvunyi

**Affiliations:** 1 Rwanda Biomedical Center, Kigali P.O. Box 7162, Rwandaayman.ame.ahmed@gmail.com (A.A.); 2 School of Public health, College of Medicine and Health Sciences, University of Rwanda, Kigali 3286, Rwanda; 3 University Teaching Hospital of Butare, Butare 15232, Rwanda; 4 School of Chemical Engineering, University of Birmingham, Edgbaston, Birmingham B15 2TT, UK; 5 School of Health Systems & Public Health, University of Pretoria, Pretoria 0002, South Africa; 6 Institute of Endemic Diseases, University of Khartoum, Khartoum 11111, Sudan

**Keywords:** prescription and use of antibiotics, integrated one health policy for antimicrobial resistance control, AMR stewardship, Gram-negative bacteria, Gram-positive bacteria, health facility-acquired infection

## Abstract

**Background**: The burden of bacterial bloodstream infections (BSIs) is rapidly increasing in Africa including Rwanda. **Methods**: This is a retrospective study that investigates the diversity, distribution, and antimicrobial susceptibility profiles of BSI bacteria in three tertiary referral hospitals in Rwanda between 2020 and 2022. **Results**: A total of 1532 blood culture tests were performed for visiting patients. Overall, the proportions of Gram-negative and Gram-positive bacteria were 48.2% and 51.8, respectively. *Staphylococcus aureus* was the predominant species accounting for 25% of all Gram-positive BSI species, and Klebsiella species represented 41% of all Gram-negative BSI species. Antimicrobial susceptibility testing revealed that Amikacin exhibited the highest activity against *Enterobacter* spp., *Serratia* spp., and *Escherichia coli* in >92% of cases and *Klebsiella* spp. in 75.7%. Meropenem and Imipenem were highly efficacious to *Salmonella* spp. (100% susceptibility), *Enterobacter* spp. (96.2% and 91.7%, respectively), and *Escherichia coli* (94.7% and 95.5%, respectively). The susceptibility of *Enterococcus* spp., *S. aureus*, and *Streptococcus* spp. to Vancomycin was 100%, 99.5%, and 97.1%, respectively. *Klebsiella* spp. was highly sensitive to Colistin (98.7%), Polymyxin B (85.6%), Imipenem (84.9%), and Meropenem (78.5%). **Conclusions**: We recommend strengthening the implementation of integrated transdisciplinary and multisectoral One Health including AMR stewardship for the surveillance, prevention, and control of AMR in Rwanda.

## 1. Introduction

Antimicrobial resistance (AMR) is a rapidly growing global and public health issue that threatens human and animal health throughout the world [1,2]. Considering that some bacterial infections are fatal for humans and animals, therefore, antibiotics are life-saving drugs, and we need to maintain their effectiveness, particularly since developing novel, safe, and effective antibiotics takes years and costs a lot of resources. Therefore, the growing burden of AMR is severely affecting case management and control of bacterial and fungal infections leading to substantial morbidity, mortality, disability, and socioeconomic burdens worldwide [3,4,5,6]. According to the World Health Organization (WHO), AMR is one of the top ten threats to global public health with alarmingly high levels of bacterial resistance observed in pathogens responsible for bloodstream infections (BSIs) [1,2,7]. Low- and middle-income countries (LMICs) are struggling the most with the control of antimicrobial-resistant bacterial pathogens involved in BSIs [1,8].

BSIs are associated with high morbidity and mortality rates worldwide, with regional and national variations in both the spectrum of causative pathogens and their resistance to available antimicrobial drugs [1,2]. For example, in China, *Escherichia coli* and *Staphylococcus aureus* were the most prevalent Gram-negative and Gram-positive bacteria responsible for BSIs, respectively [8,9]. Similarly, European surveillance networks, such as the European Antimicrobial Resistance Surveillance Network (EARS-Net), have identified *E. coli* and *Staphylococcus aureus* as the predominant pathogens involved in BSIs [9]. Other regions exhibit distinct trends such as in South Korea mainly reporting *Streptococcus* spp. and *Klebsiella* spp. alongside *E. coli* and *Staphylococcus aureus* [10] as predominant, while in Japan, *Streptococcus* spp. and *Klebsiella* spp. are reported as prominent BSI pathogens [11]. In Malawi, a 19-year cohort study revealed non-typhoidal *Salmonella*, *Salmonella typhi*, and *Streptococcus pneumoniae* as the leading of BSIs [12]. A recent Global Burden of Disease (GBD) study has estimated that 1.05 million deaths were associated with AMR in Africa in 2019, with 5.3% of them associated with BSIs [1]. Sub-Saharan Africa (SSA) bears the brunt of AMR mortality, with a rate of 23.5 deaths per 100,000 persons in 2019 [1,13]. Eastern Africa ranked second following SSA, with a rate of 21.4 deaths per 100,000. Several factors contribute to this disproportionate burden of AMR in SSA, including poverty, self-medication, inadequate regulation or stewardship of antimicrobial use, and limited access to effective alternatives when resistance is identified [14].

Rwanda has witnessed a concerning rise in the prevalence of AMR among bacterial infections. A recent study conducted in Rwandan referral hospitals revealed high resistance rates to commonly prescribed antibiotics for BSIs [15]. Rates of resistance in Gram-negative isolates to Penicillin, Trimethoprim/Sulfamethoxazole, Ampicillin, and Ceftriaxone were about 92%, 83%, 82%, and 76%, respectively. These high resistance rates were associated with factors such as prior attendance to multiple healthcare facilities, recent surgery or antibiotic exposure, and hospital-acquired infection [15].

However, data on BSIs and their profiles of resistance/susceptibility to antibiotics in Rwanda and the surrounding region remain largely limited. This severe lack of up-to-date evidence is hindering policymaking, strategic planning, and the implementation of cost-effective interventions to prevent and control AMR, particularly among BSIs. Therefore, this study aims to fill this gap in knowledge by investigating retrospective records in major national hospitals in Rwanda to shed some light on the issue of AMR among BSIs in the country.

## 2. Results

**Figure 1:**
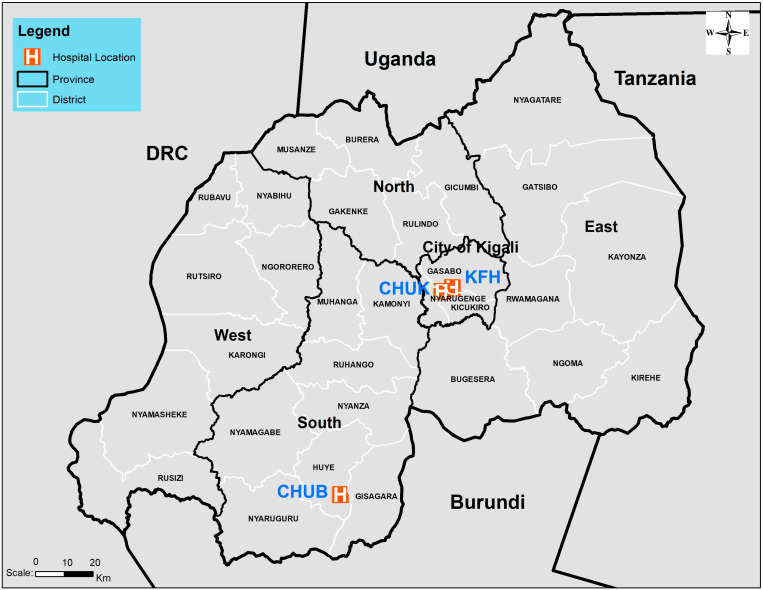
The map of Rwanda shows the geographical location of the three major hospitals in which this study was implemented.

A record of 1532 blood cultures and their results from three major national hospitals were available for this analysis (Figure 1). Data were extracted from hospital records in the King Faisal Hospital (KFH), which had the highest number of positive cultures with 822 (54%), followed by the University Teaching Hospital of Kigali (CHUK) with 539 (35%), and the University Teaching Hospital of Butare (CHUB) with 171 (11%) (Figure 1 and Table 1).

Annual distribution of the BSIs was almost stable throughout the three years of the study around an average of 510 ± 50 cases with a slight increase in 2021. Investigating the distribution of bacterial pathogens through the three main study sites/healthcare facilities revealed significant variation (Table 1). Significant variation was observed in the distribution of Gram-negative bacteria; *Klebsiella* spp. was the most prevalent bacterial infection across the three hospitals resulting in about 41% of the overall reported infections. It was responsible for 32% of infections reported from KFH, followed by *E. coli* and *Acinetobacter* spp. each accounting for about 14% of the infections (Table 1), while, in CHUK, *Klebsiella* spp. was involved in almost half (46%) of the reported infections, followed by *E. coli* and *Acinetobacter* spp. contributing about 24% and 16% in the incidence of bacterial infections, respectively. On the other hand, CHUB reported 45% *Klebsiella* spp., 17% *Acinetobacter* spp., and only 11% *E. coli*. Meanwhile, *Salmonella* spp. was a main issue in CHUB reporting 14% prevalence, and *Serratia* spp. was only reported from KFH (6%) (Table 1).

A similar significant variation was observed in the distribution of the Gram-positive bacteria pathogens where *Staphylococcus aureus* (50%) and *coagulase-negative Staphylococci (CNS)* (35.6%) were the most common causative agents (Table 1). However, most bacterial infections in KFH were caused by *CNS* (50%) followed by *S. aureus* (33%%). In contrast, the majority of infections in CHUK were caused by *S. aureus* (94%). In CHUB, over half of the bacterial infections were caused by *S. aureus* (56%), 28% were caused by *CNS,* and interestingly, 15% were due to *Streptococcus* spp. infections (Table 1).

To identify patients or population groups at high risk of BSIs, we further analyzed the distribution of different BSI pathogens by the medical department where the patients were presented and requested the blood culture test. Most of the Gram-negative bacteria were from pediatric (23%), ICU (20%), and internal medicine (16%) departments (Table 2). For Gram-positive bacteria, the majority were from emergency (23%), pediatric (18%), internal medicine (17%), and ICU (17%) departments (Table 2).

**Figure 2:**
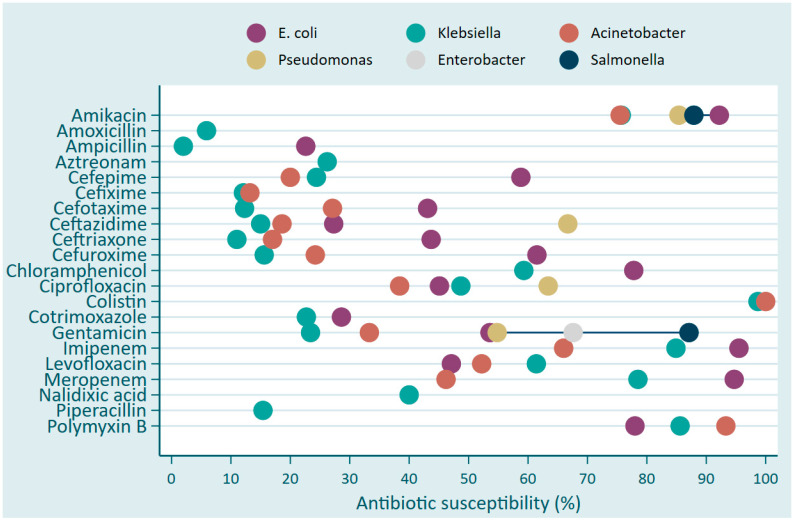
The proportion of antibiotic susceptibility of Gram-negative pathogens associated with bacterial bloodstream infections in Rwanda between 2020 and 2022.

*Klebsiella* and *Acinetobacter* ssp. showed predominantly high resistance with less than 30% of the isolates susceptible to various antibiotics including Amoxicillin, Ampicillin, Aztreonam, Cefepime, Cefixime, Cefotaxime, and Ceftazidime, as well as Ceftriaxone, Cefuroxime, Cotrimoxazole, Gentamicin, and Piperacillin (Figure 2). *E. coli* and *Klebsiella* exhibited high susceptibility towards Colistin, Amikacin, Polymyxin B. Meropenem, and Imipinem, ranging around 90% and 80%, respectively, while Acinetobacter was less susceptible for Imipinem (67%) and for Meropenem (45%) (Figure 2).

**Figure 3:**
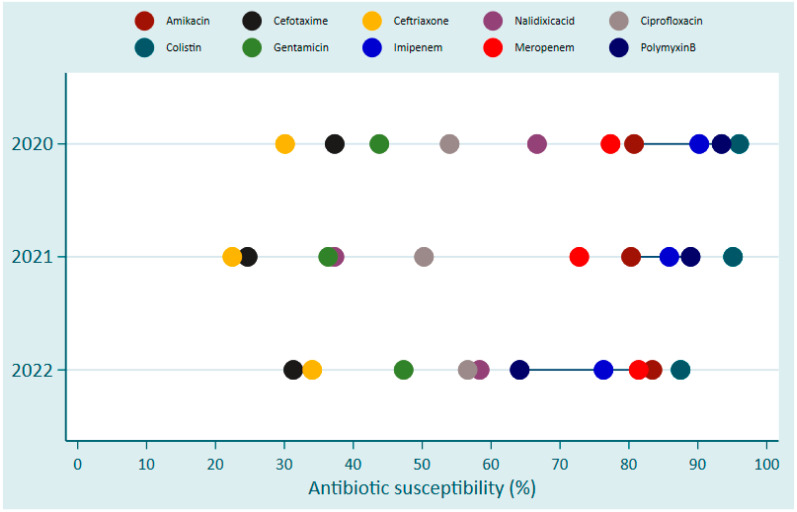
AMR development among Gram-negative bacterial bloodstream infection pathogens in Rwanda between 2020 and 2022.

Despite the increased susceptibility toward Colistin, Polymyxin B, Imipinem, Amikacin, and Meropenem among Gram-negative bacteria causing BSIs, a trend in the development of resistance against Colistin was observed. This was indicated by the gradually decreasing susceptibility from 96% (48/50) in 2020 and 95% (97/102) in 2021 to 87.5% (35/40) in 2022 (Figure 3). Similar trends were observed for Polymyxin B where susceptibility declined from 93.4% in 2020 and 88.9% in 2021 to 64.1% in 2022 (Figure 3).

**Figure 4:**
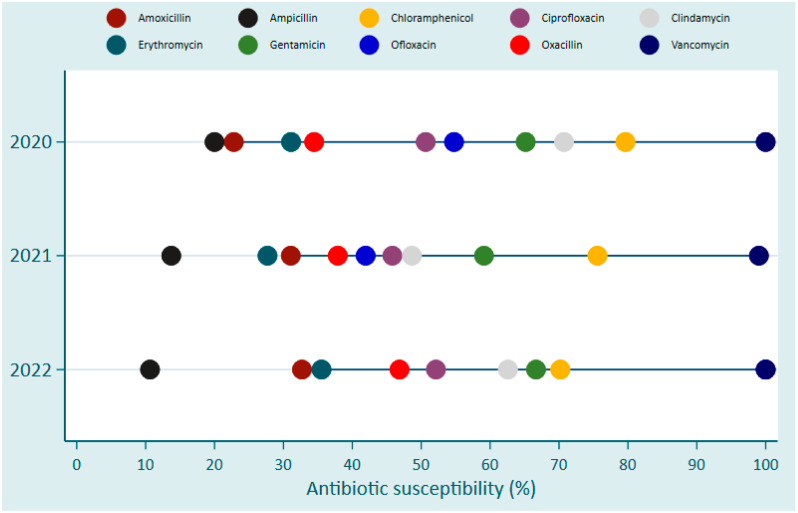
AMR development among Gram-positive bacterial bloodstream infection pathogens in Rwanda between 2020 and 2022.

Overall, Vancomycin and Chloramphenicol were the two most effective antibiotics over the 3-year period against Gram-positive bacteria with a sensitivity of over 90% and 70–80%, respectively (Figure 4).

**Figure 5:**
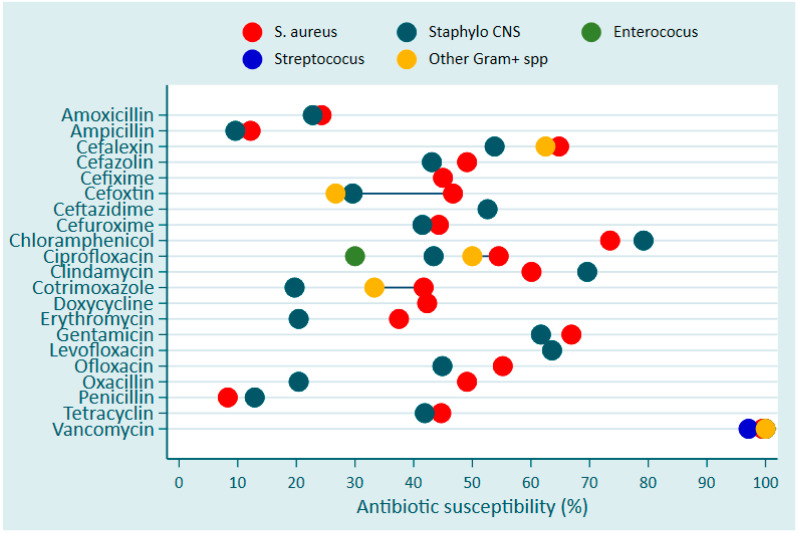
Susceptibility proportion of Gram-positive bacteria associated with bacterial bloodstream infections to commonly used antibiotics in Rwanda between 2020 and 2022.

Vancomycin was the only antibiotic that exhibited a nearly full susceptibility followed by Chloramphenicol, which showed a susceptibility level between 70% and 80% for *S. aureus* (Figure 5). The effectiveness of other antibiotics including Gentamicin, Cephalexin, and Clindamycin on *S. aureus* was in the range between 60 and 70% (Figure 5).

## 3. Discussion

AMR is a major threat to the effectiveness of antibiotics, which are crucial for treating many potentially fatal infections including BSIs, TB, and lower respiratory infections [1,2,6,16]. This issue is particularly pressing in LMICs including Rwanda, where limited treatment options are available for the case management of bacterial and fungal infections [1,15]. This research highlights the diversity of pathogens involved in BSIs in Rwanda between 2020 and 2022, their distribution among patients treated for different medical conditions, and their susceptibility to the main antibiotics. We observed a high diversity of pathogens involved in the development of bacterial BSIs among patients hospitalized in three major hospitals in Rwanda, with over 20 species of Gram-negative bacteria and around 10 species of Gram-positive bacteria isolated. *S. aureus*, *Klebsiella* spp., and *Staphylococcus (CNS)* were the most prevalent pathogens (65%), contributing to 26%, 20%, and 19% of the infections, respectively. These findings align with regional studies, highlighting *S. aureus* and *Klebsiella* spp. as leading causes of BSIs in the WHO African region [13]. However, *Staphylococcus (CNS)* also known as *coagulase-negative Staphylococcus* is commonly understudied because it is considered part of the commensal flora or microbiome of the skin [17]. Nevertheless, it is reported as among the top prevalent causes of BSIs in high-income countries [18]. It has recently been reported to constitute about 34% of the BSIs in Morocco [17]. However, this might be attributed to poor management of wounds that resulted in opportunistic infections.

The majority of positive cultures were from pediatric, ICU, and internal medicine departments, suggesting a wider exposure among the local population to bacterial pathogens. This concurs with previous findings from Rwanda, Ethiopia, and Ghana [7,15,19]. However, the high prevalence among children could be attributed to the fact that children and, in particular, neonates are more prone to BSIs mainly due to their weak immunity [20]. Also, in LMICs, children are more affected by infections because of poor sanitation and other environmental factors associated with low socioeconomic living conditions. On the other hand, the prevalence (15%) of BSIs among patients in the ICU might be due to the delay in seeking healthcare resulting in high admission rates to the ICU because of a severe bacterial infection or a health-facility-acquired infection due to poor implementation of effective infection prevention and control [21,22],. Nevertheless, further research is needed to investigate the actual burden of BSIs among patients arriving in the ICU and the risk factors associated with BSIs in ICU wards. Meanwhile, internal medicine wards are mostly occupied by old patients who struggle with comorbidities or live in settings with inadequate hygiene and sanitation increasing their risk and vulnerability to acquiring BSIs [23,24].

Wide variation was observed in the susceptibility of different BSI pathogens to different antibiotics over the three-year period of the study. *E. coli* showed high sensitivity toward Amikacin and Imipenem in 2020, while *S. aureus* remained predominantly sensitive to Vancomycin throughout 2020–2022. However, in general, resistance to Meropenem and Imipenem was noticed among Gram-negative bacteria; similar scenarios were reported in North America and Asia [25]. Unfortunately, both antibiotics, Meropenem and Imipenem, belong to the carbapenem class, which is considered the last resort for treating multidrug-resistant pathogens [26,27]. Resistance to carbapenem antibiotics is of great concern as there are limited alternative antibiotics that could be used against carbapenem-producing Enterobacteriaceae [28]. Furthermore, resistance to Colistin and Polymyxin B was recorded, and both antibiotics belong to the Polymyxin class, which targets the lipopolysaccharide (LPS) membrane of Gram-negative bacteria resulting in death due to the destruction of LPS and leakage of cellular content [29].

The temporal analyses of AMR show a rapid change in trends in pathogen-specific drug resistance. Interestingly, this change in the antibiotic-resistance profiles of different species of bacteria is not uni-directional. This suggests that through implementing proper antimicrobial stewardship strategies and interventions, the effectiveness of some of these drugs might be restored over time in the population of some pathogens due to the evolutionary heavy cost of maintaining resistance genes without continuous exposure [29]. For instance, a concerning trend in rapidly growing and spreading resistance to commonly used antibiotics was noticed, particularly among *Klebsiella* spp. and *Acinetobacter* ssp.; unfortunately, both are associated with higher mortality rates. Drug-resistant *S. aureus*, *Klebsiella* spp., and *E. coli* have been associated with high mortality rates owing to the complexity involved in their clinical management and treatment [1,2,13]. This emphasizes the critical need for the implementation of the Rwanda National Action Plan on Antimicrobial Resistance (NAPAMR) to regulate antibiotic usage, prevent the spread of bacterial AMR, and improve both case management and infection control in the country. Achieving these objectives will necessitate a multi-sectoral collaboration led by health authorities from various domains, including human, animal, and environmental health sectors.

This high diversity of bacterial pathogens involved in BSIs and the growing resistance to currently available antibiotics are of significant public health importance. Therefore, there is an urgent need to strengthen the surveillance, prevention, and control of AMR in the country comprehensively through the implementation of a transdisciplinary multisectoral integrated One Health strategy [30,31,32,33]. Advanced technology such as the use of Artificial Intelligence (AI) might improve the cost-effectiveness of surveillance, prevention, and control measures through early prediction, monitoring of population dynamics, and enhancement of the diagnostic capacity by integrating AI-powered diagnostic algorithms [34]. Furthermore, additional investment is needed to strengthen the implementation of the guidelines of the World Health Organization (WHO) for infection prevention and control in healthcare facilities to reduce the burden of healthcare facility-acquired infection [22,35,36]. Considering the high risk, restrictive measures and international guidelines must be followed to investigate, prepare, prevent, and control multidrug-resistant (MDR), extensively drug-resistant (XDR), and pandrug-resistant (PDR) bacteria in Rwanda and the surrounding region [37].

One major limitation of this study is that it relies on analyzing secondary programmatic data that were collected through routine surveillance in three hospitals. Therefore, variations in the diagnostic capacity, performance, and expertise of the attending microbiologists and the protocol they followed might unintentionally introduce some bias in the reported results. This is mainly indicated by the variation in the protocols for processing the samples in the different hospitals. Nevertheless, the currently reported results correspond with previous findings from the country suggesting the insignificance of this variation [38]. However, we recommend integrating the surveillance of AMR in the country into a single robust standardized system to avoid such potential bias.

In response to this growing threat, Rwanda through the Rwanda Biomedical Centre (RBC) has invested in multidimensional strategic interventions to counteract AMR and the associated reduction in antibiotic effectiveness. This includes the expansion of the integrated surveillance and response system to be implemented throughout the country and includes veterinary surveillance under a multisectoral “Rwanda National Antimicrobial Resistance Surveillance Operational Plan 2024–2028”. Furthermore, RBC is currently piloting the implementation of Wastewater-Based Epidemiology supported by genomic surveillance to monitor and track the dynamic of infectious diseases and AMR at the community level. Moreover, these strategic interventions will be supported by the establishment of a Biotechnology Centre in the country to lead the innovation, development, and repurposing of novel and existing drugs and vaccines. These strategies and interventions are mainly tailored to low-resource settings like Rwanda and other African countries.

## 4. Materials and Methods

### 4.1. Study Design and Study Sites

This was a retrospective descriptive study of blood cultures processed in laboratories of three main national tertiary referral hospitals in Rwanda from January 2020 to August 2022. These hospitals include CHUK, CHUB, and KFH, and they serve as the central network for national AMR surveillance. CHUK is located in the Nyarugenge district of Kigali City and is the biggest referral hospital in Rwanda with a capacity of 519 beds, CHUB is situated in the Huye District of the Southern Province with a capacity of 500 beds, and KFH is located in Kigali City and is the largest private and university teaching hospital in Rwanda with 160 beds (Figure 1).

### 4.2. Data Collection and Laboratory Methods

Blood culture isolates and corresponding antimicrobial susceptibility profiles were collected from laboratory registers used in routine clinical care. These microbiological data were linked to clinical data by serial numbers. Blood samples were obtained where clinically indicated, using aseptic methods by trained healthcare staff, and inoculated to BD BACTEC (Becton-Dickinson, Franklin Lakes, NJ, USA) bottles. Blood samples were sent to the bacteriology laboratory and incubated in the BACTEC FX automated system for five days as per the standard blood culture protocol. Gram staining was performed on blood culture signaling positive growth. Subcultures onto blood agar (BA) and MacConkey agar (MCA) were performed for Gram-positive and Gram-negative isolates, respectively, and in accordance with standardized operating procedures, followed by biochemical testing using Analytical Profile Indexing (API-20). At CHUK and KFH, Phoenix M50 (Becton-Dickinson, Franklin Lakes, NJ, USA) was used for bacterial identification in accordance with the manufacturer’s instructions. CHUB used the Kirby–Bauer disk diffusion method for antibiotic susceptibility testing. Here, the turbidity of the normal saline bacterial suspension was adjusted to match the 0.5 McFarland standards. Mueller–Hinton agar was used and incubated aerobically at 37 °C for 16–18 h. Inhibition zones were measured and interpreted as sensitive or resistant based on Clinical and Laboratory Standards Institute (CLSI) breakpoints. For quality management, the following reference strains (ATCC25923, 25922, 27852, 49619, or 49247) were used as controls.

### 4.3. Data Management and Statistical Analysis

Data from laboratory records were entered into the Demography and Health Surveys (DHS) platform and extracted into Microsoft Excel 2016. Descriptive statistics (frequency and proportions of AMR) were computed using Stata software version 4.1.3 to investigate the distribution of clinical and biological characteristics. The susceptibility level of isolated pathogens was reported for all antibiotics while pathogens with less than 30 isolates were excluded in the AMR analysis.

## 5. Conclusions

This study showed the bacterial pathogens associated with BSIs and their antimicrobial susceptibility profiles at three national tertiary referral hospitals in Rwanda. *K. pneumoniae*, *E. coli*, *A. baumannii*, and *S. aureus* were among the predominant pathogens associated with BSIs. Antibiotic susceptibility testing results show that clinically relevant drug-resistant BSI bacteria are prevalent in the studied population with resistance to carbapenem antibiotics and Polymixin class antibiotics also noted, raising concerns about the limited alternatives for treating multidrug-resistant pathogens. This underscores the urgent need for strengthening antimicrobial stewardship programs and adopting a surveillance system to combat the emergence of AMR.

## Figures and Tables

**Table 1:** The diversity and distribution of Gram-negative and -positive bacterial pathogens by hospital between 2020 and 2022.

Pathogen Species	King Faisal Hospital (KFH)		Kigali University Teaching Hospital (CHUK)		Butare University Teaching Hospital (CHUB)		Total		
Gram-negative	*n*	%	*n*	%	*n*	%	*n*	%	*p*-value
*Escherichia coli*	38	13.7	78	23.8	15	11.4	131	17.8	<0.001
*Klebsiella* spp.	89	32.0	152	46.3	59	44.7	300	40.7	
*Acinetobacter* spp.	38	13.7	51	15.6	22	16.7	111	15.0	
*Pseudomonas* spp.	28	10.1	12	3.7	8	6.1	48	6.5	
*Enterobacter* spp.	33	11.9	9	2.7	5	3.8	47	6.4	
*Salmonella* spp.	6	2.2	18	5.5	18	13.6	42	5.7	
*Serratia* spp.	17	6.1	0	0.0	0	0.0	17	2.3	
* *Other* spp.	29	10.4	8	2.4	5	3.8	42	5.7	
**Total**	**278**	**100**	**328**	**100**	**132**	**100**	**738**	**100**	
Gram-positive	*n*	%	*n*	%	*n*	%	*n*	%	*p*-value
*Staphylococcus aureus*	177	32.5	198	93.8	22	56.4	397	50.0	<0.001
*Staphylococcus (CNS)*	269	49.5	3	1.4	11	28.2	283	35.6	
*Enterococcus* spp.	32	5.9	4	1.9	0	0.0	36	4.5	
*Streptococcus* spp.	24	4.4	6	2.8	6	15.4	36	4.5	
** *Other* spp.	42	7.7	0	0.0	0	0.0	42	5.3	
**Total**	**544**	**100**	**211**	**100**	**39**	**100**	**794**	**100**	

* Other spp.: *Proteus* spp. (9), *Coccobacilli* (8), *Citrobacter* spp. (4), *Providencia* spp. (4), *Neisseria* spp. (4), *Morganella* spp. (3), *Cocci* (3), *Micrococcus* spp. (1), *Stenotrophomonas* spp. (1), *Rods* (1), *Anaerobic coccobacilli* (1), *Haemophilus haemolyticus* (1), and *Moraxella* spp. (1). ** Other spp.: *Cocci* (41) and *Coccobacilli* (1).

**Table 2:** The distribution of the detected Gram-negative and -positive species of bacterial bloodstream infection pathogens by hospital department.

Pathogen Species	OPD		Internal Medicine		Surgery		Emergency		ICU		Pediatrics		Gynecology		Neonatology		NICU		Total		
*Gram −*	*n*	%	*n*	%	*n*	%	*n*	%	*n*	%	*n*	%	*n*	%	*n*	%	*n*	%	*n*	%	*p*-value
*Escherichia coli*	11	24.4	36	30.0	7	24.1	19	22.6	15	10.2	27	16.0	9	81.8	6	7.1	1	3.6	131	17.8	<0.001
*Klebsiella* spp.	7	15.6	36	30.0	12	41.4	31	36.9	56	38.1	82	48.5	1	9.1	54	64.3	15	53.6	300	40.7	
*Acinetobacter* spp.	10	22.2	13	10.8	5	17.2	9	10.7	32	21.8	23	13.6	0	0.0	11	13.1	1	3.6	111	15.0	
*Pseudomonas* spp.	3	6.7	8	6.7	4	13.8	4	4.8	10	6.8	12	7.1	0	0.0	4	4.8	1	3.6	48	6.5	
*Enterobacter* spp.	2	4.4	13	10.8	0	0.0	4	4.8	13	8.8	6	3.6	0	0.0	4	4.8	2	7.1	47	6.4	
*Salmonella* spp.	9	20.0	8	6.7	0	0.0	9	10.7	0	0.0	15	8.9	1	9.1	0	0.0	0	0.0	42	5.7	
*Serratia* spp.	2	4.4	0	0.0	0	0.0	3	3.6	5	3.4	0	0.0	0	0.0	0	0.0	5	17.9	17	2.3	
*Other* spp.	1	2.2	6	5.0	1	3.5	5	6.0	16	10.9	4	2.4	0	0.0	5	6.0	3	10.7	42	5.7	
**Total**	**45**	**100**	**120**	**100**	**29**	**100**	**84**	**100**	**147**	**100**	**169**	**100**	**11**	**100**	**84**	**100**	**28**	**100**	**738**	**100**	
*Gram +*	*n*	%	*n*	%	*n*	%	*n*	%	*n*	%	*n*	%	*n*	%	*n*	%	*n*	%	*n*	%	*p*-value
*Staphylococcus aureus*	30	60.0	76	55.5	16	59.3	98	53.0	46	34.3	82	58.6	6	40.0	26	78.8	10	23.3	397	50.0	<0.001
*Staphylococcus (CNS)*	19	38.0	43	31.4	4	14.8	62	33.5	64	47.8	38	27.1	8	53.3	5	15.2	19	44.2	283	35.6	
*Enterococcus* spp.	0	0.0	5	3.7	2	7.4	10	5.4	15	11.2	1	0.7	0	0.0	0	0.0	3	7.0	36	4.5	
*Streptococcus* spp.	0	0.0	6	4.4	3	11.1	6	3.2	3	2.2	11	7.9	0	0.0	1	3.0	4	9.3	36	4.5	
*Other* spp.	1	2.0	7	5.1	2	7.4	9	4.9	6	4.5	8	5.7	1	6.7	1	3.0	7	16.3	42	5.3	
**Total**	**50**	**100**	**137**	**100**	**27**	**100**	**185**	**100**	**134**	**100**	**140**	**100**	**15**	**100**	**33**	**100**	**43**	**100**	**794**	**100**	

Abbreviation: OPD = Outpatient Department, ICU = Intensive Care Unit, and NICU = Neonatal Intensive Care Unit.

## Data Availability

All data produced in this study are included in the published paper.
